# Immune Response Regulation by Antigen Receptors’ Clone-Specific Nonself Parts

**DOI:** 10.3389/fimmu.2018.01471

**Published:** 2018-06-25

**Authors:** Hilmar Lemke

**Affiliations:** Biochemical Institute of the Medical Faculty, Christian-Albrechts-University at Kiel, Kiel, Germany

**Keywords:** B cell antigen receptor (BCR), T cell antigen receptor (TCR), adaptive immune response, regulation, idiotype, self–nonself, immunogenicity, transgenerational imprinting

## Abstract

Antigen determinants (epitopes) are recognized by the combining sites (paratopes) of B and T cell antigen receptors (BCR/TCR), which again express clone-specific epitopes (idiotopes) that can be recognized by BCR/TCR not only of genetically different donors but also within the autologous immune system. While xenogeneic and allogeneic anti-idiotypic BCR/TCR are broadly cross-reactive, only autologous anti-idiotypes are truly specific and of functional regulatory relevance within a particular immune system. Autologous BCR/TCR idiotopes are (a) somatically created at the third complementarity-determining regions, (b) through mutations introduced into BCRs during adaptive immune responses, and (c) through the conformational impact of both. As these idiotypic characters have no genomic counterparts they have to be regarded as antigen receptor-intrinsic nonself-portions. Although foreign, however, they are *per se* non-immunogenic, but in conjunction with immunogenicity- and adjuvanticity-providing antigen-induced immune responses, they induce abating regulatory idiotypic chain reactions. The dualistic nature of antigen receptors of seeing antigens (self and nonself alike) and being nonself at the same time has far reaching consequences for an understanding of the regulation of adaptive immune responses.

## The Clonal Selection Theory—Keystone of Immunology

The clonal selection theory put forward almost 60 years ago (1) has developed into the clonal selection law (CSL) (2) that is widely accepted as keystone and current paradigm of adaptive immunity and immunological thinking in general (3–5). The core features of CSL comprise that (i) membrane-bound antigen receptors of B cells (BCR) and T cells (TCR) are generated in an antigen-independent genetic process, (ii) these preformed receptors are specific for particular antigens and thus mediate the specific clonal selection of the relevant cells encountering the corresponding antigen during microbial infections or experimental immunizations, and (iii) depending on antigen parameters like quantity, quality [various parameters determining its immunogenicity including size, structure, ability to induce cytokine responses after binding to non-clonal receptors on antigen-presenting cells, presentability of antigen fragments *via* major histocompatibility complex (MHC) molecules, etc.], route of entry, place of action in tissues, presence of natural microbial or admixed adjuvants, and the genetic constitution of the host (2, 6) such interactions may either lead to activation and proliferation of the respective B and T cells that execute the ensuing immune response (leading to differentiation into end stage effector and memory cells) or to the induction of tolerance by a variety of mechanisms. It is of historic interest that in 1955, Jerne (7, 8) proposed that natural antibodies (nAbs) make the first encounter with antigen and thus support a theory of antibody-selection driven adaptive immune response (9).

## Antigen-Specific Initiation and Non-Specific Progression of Adaptive Immune Responses?

Thus, BCR and TCR mediate the *initiation* of adaptive immune responses. While cell-bound BCR directly react with native antigens, the diverse subpopulations of naïve T cells like CD8^+^ cytotoxic cells and a multitude of helper and suppressor CD4^+^ T cells [T_H_1, T_H_2, T_H_17, follicular helper T cells (T_FH_), and regulatory T cells (Tregs)] (10) can only participate in adaptive responses after antigen has been taken up and degraded by various antigen-processing cells which then present antigen fragments on MHC molecules on the cell surface. Depending on the nature of the antigen, immune responses develop along different pathways. The initial encounter of BCR/TCR with thymus-dependent (TD) antigens, in particular hapten-coupled proteins as model antigens, induce partial activation, proliferation, differentiation, and migration through secondary lymphoid organs and cellular interactions beginning at extrafollicular sites and continuing in B cell follicles with the establishment of germinal centers (GCs) [reviewed in Ref. (11–15)]. In the GCs, B cells undergo extensive T_FH_-dependent proliferation and somatic hypermutations (SHM) that are accompanied by class-switch recombination (CSR) and immune maturation through selection of higher affinity clones. The GC responses not only lead to differentiation of class-switched memory B cells that participate in secondary responses since they can be activated in the presence of humoral antibody (16) but also to long-lived plasma cells that secrete high amounts of antibody independently of antigen and to memory B cells (17). By contrast, immune responses to complex antigens like *Salmonella typhimurium* can also develop independently of GC formation in follicles and extrafollicular sites and this response is also accompanied by CSR and SHM-mediated immune maturation (18). Even the heterogeneous populations of memory B cells may be generated in as well as outside GCs (19) and may retain IgM BCR on their cell surface (20). In addition, a special B cell memory can also be induced by thymus-independent type I (TI-1—mitogenic) and type II (TI-2—polymeric) antigens (21) and this type of memory is related to antigen-specific IgG antibodies (22, 23).

The complex events of cellular interactions, proliferation, differentiation, and migration during progression of adaptive immune responses through the different specialized microenvironments of secondary lymphoid organs are studied by associating the involved cell lineages with the expression of transcription factors, activation and differentiation markers, cytokine and chemokine secretion, and expression of the respective membrane receptors [reviewed in Ref. (11–13, 19, 24)]. The main driving force during GC differentiation is supposed to depend on the selection of higher affinity clones that are generated by SHM (25). However, in response to two complex antigens (Bacillus anthracis protective antigen and influenza hemagglutinin) half of the GC B-cells did not bind the antigen used for immunization despite showing signs of activation, namely biased usage of VH genes, exhibition of mutations, and clonal proliferation similar to antigen-binding B cells (26). Also in the initial extrafollicular response to the complex antigen *S. typhimurium*, only a small fraction of B-cells showed specific binding to the antigen and underwent SHM-driven immune maturation while the majority of cells did not bind antigen (18). Although these antigen non-specific responses are not understood, it currently appears to be generally accepted that, after antigen-dependent BCR/TCR-specific *initiation* of adaptive immune responses, all further steps of B and T cell differentiation that depend on the *initial* clonal selection are associated with and can be described by the expression of *non*-clonal and antigen-*non*-specific factors as mentioned above.

**Figure 1:**
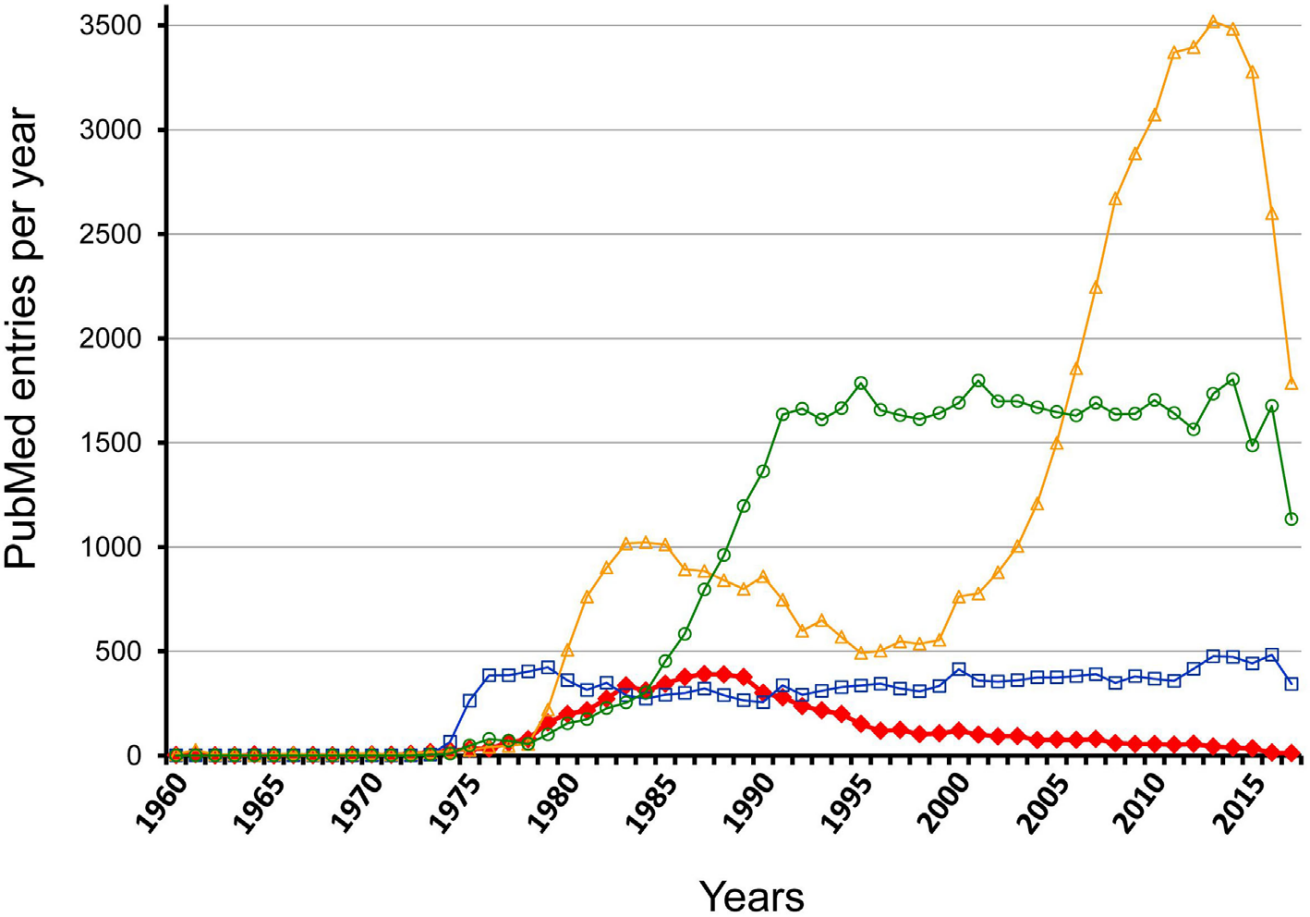
Comparison of PubMed entry “*idiotype*” with related marks per year between 1960 and 2017. The figure shows the results of a PubMed search for the following keywords: idiotype—red filled rhombus; regulatory T cell—yellow open triangles; B cell antigen receptor—blue open squares; T cell antigen receptor—light green open circles.

However, this view completely ignores investigations made some decades ago, which proclaimed that the antigen-specific initiation of the response is followed by a cascade-like chain reaction directed at clonotypic/idiotypic determinants in the variable regions of BCR and TCR. These investigations had led to the hypothesis of an idiotypic network that is functionally active before any encounter of environmental antigens and involved in regulation of immune responses as well (27). Publication rates for the term “*idiotype*” reveal this temporary interest that increased 1978–1988 and decreased to a low level during the next decade while related keywords, as for instance, “*B cell antigen receptor*” and “*T cell antigen receptor*” document a prolonged and more constant interest; however, for “*regulatory T cell*” a rather dramatic change in interest is visible (Figure 1). Investigations on idiotypic regulation have been viewed as “*tidal wave*” that “*has receded leaving behind an empty beach*” (28). Accordingly, idiotypic research once regarded as the cutting edge of immunology is nowadays even eliminated from most textbooks that only denote the term “*idiotype*” without ascription of any functional relevance (4, 5). A detailed description of this “*Rise and Fall of a Scientific Paradigm*” has been composed by Eichmann (29) in which he wondered (p. 3): “*How can such a thing happen? How is it possible that hundreds of scientists engage in work, over periods of more than a decade, that thereafter gets disposed as meaningless*?” Ironically enough, despite this condemnation his work also contains statements from 11 well-known former idiotype-researchers as key-witnesses of this time that “*an immune network exists*” (p. 157) and “*idiotype-based regulations exists*” (p. 161) and that it “*was all solid work*” (p. 166). This is an extremely disappointing situation calling for a solution; it certainly does not allow idiotypic research on regulation to be simply filed away as if it had never happened. A recent review of the current understanding of idiotypic research summarizes the expectation and return of studies on the idiotypic network (30).

## Principle Reactions in Idiotypic Regulations

In 1963, antigen-induced antibodies themselves were found to be immunogenic and can induce anti-antibodies (31, 32) that reacted with specific determinants of the inducing antibodies (idiotypes or Ab1) and so were designated anti-idiotypes (Greek ιδιοσ, unique). Anti-idiotypic (Ab2) antibodies represent a heterogeneous group recognizing different types of idiotypic determinants (idiotopes) and are classified on the basis of their reactivity with Ab1 idiotopes in variable distances from the antigen combining site (paratope) [reviewed in Ref. (33)]. (a) Ab2α react with idiotopes located outside the paratope in vicinity to and in framework regions and their binding to Ab1 cannot be inhibited by antigen. (b) Although another set of Ab2 (Ab2γ) recognize paratope-associated idiotopes, their binding to Ab1 can also not be inhibited by antigen. (c) Anti-idiotypic antibodies of the Ab2β type react with idiotopes that overlap or even may supposedly coincide with the Ab1 paratope. The antigen-inhibitable reactivity of these Ab2β has been taken as indication that the paratopes of particular Ab2β resemble the antigenic epitope and thus may carry an internal images of it (34). Hence, this type of Ab2 can be employed as surrogate antigen (35, 36). However, structural analyses have shown that the mimicking of antigenic epitopes is functional and mediated by similar binding interactions, but does not depend on identical 3D structures (37). This nonstructural but functional mimicry can even be observed with anti-idiotypic antibodies reacting with Ab1 to nonprotein antigens. For instance, although Ab2 are certainly not able to mimic a polysaccharide structure they may induce a polysaccharide-specific response to the related antigen (38, 39). Hence, since true internal images of external epitopes seemingly do not exist, it has been proposed to replace the “internal image” concept as well as the designations for the different Ab2 subtypes by be term “network antigens” (40). (d) A fourth type of anti-idiotypes are Ab2ε antibodies or epibodies which not only display an Ab1-directed anti-idiotypic reactivity but also react with an epitope of the related antigen (41). Interestingly, Ab2 antibodies that are similar to but distinct from Ab2ε have been described, which not only exhibit Ab1-binding anti-idiotypic but also antigen-binding as well as self-binding activities (42, 43).

Contrary to their nomination, idiotypes not only carry clone/individually specific (IdI) but also cross-reactive idiotopes. On the one hand, such cross-reactive idiotopes (IdX or CRI) have been observed on antibodies exhibiting a particular antigen-specificity as shown in the antibody response of BALB/c mice to the random synthetic terpolymer of (Glu^60^Ala^30^Tyr^10^)n (GAT) that are characterized by the cross-reactive or public idiotype pGAT, which is detected with a xenogeneic rabbit antiserum (44). Likewise, antibodies reacting with the hapten p-azobenzenearsonate (ABA) can exhibit different families of CRIs (45). On the other hand, idiotypic cross-reactivity is even exhibited by antibodies that are stimulated by different external antigens. Already in 1971 it was observed that idiotypic specificities could be expressed by immunoglobulins with different binding specificities (46). For instance, murine 2,4-dinitrophenyl (DNP) antibodies carry the Id460-idiotype which is also expressed on antibodies reacting with the mouse pathogen Pasteurella pneumotropica (47). A particular idiotope (B5^+^) of anti-dextran antibodies in BALB/c mice is expressed on a subset of antibodies from A/J mice that react with the hapten NIP (48). An idiotope of a BALB/c levan-specific myeloma protein that is defined by a syngeneic monoclonal Ab2 is expressed on monoclonal antibodies of different mouse strains that are encoded by V region genes other than the corresponding myeloma and react with different nonself- and self-antigens (49). Moreover, common idiotopes have also been detected on anti-DNA auto-antibodies binding to different autoantigens (50).

Anti-idiotypic responses can not only be induced to antigen-reactive Ab1 but also to anti-idiotypic Ab2 antibodies. For instance, such anti-anti-idiotypic antibodies (Ab3) have been induced in rabbits that have been immunized with a polyclonal preparation of Ab2 which were induced with purified hyperimmune Ab1 reacting with the carbohydrate of *Micrococcus lysodeikticus* bacteria (51). Although these Ab3 shared idiotypic specificities with the corresponding Ab1, they did not react with the antigen. However, when these Ab2-immunized Ab3-producing rabbits were immunized with *M. lysodeikticus*, they produced anti-carbohydrate antibodies (Ab1′) that idiotypically resembled Ab1. Hence, the composition of the antigen-activated repertoire depends on the idiotypic history of an individual. This conclusion has been corroborated, among others, in a syngeneic experimental system in BALB/c mice, which were immunized with a levan-binding myeloma protein. These mice not only produced Ab2 but also Ab3 antibodies which could further be applied to induce anti-anti-anti-idiotypes (Ab4) (52). The members of this idiotypic cascade are idiotypically connected in that Ab3 and Ab1′ share a cross-reactive regulatory idiotope that reacts with both Ab2 and Ab4. In addition, the immune response to the related antigen levan could be variably influenced by the state of activation of Ab2, Ab3, and Ab4 of this idiotypic cascade (52). Such Ab2-reactive regulatory idiotopes on nonprotein antigens have also been observed in responses to steroid hormones, ligands, or drugs and glycolipids [reviewed in Ref. (33)]. Regulatory idiotopes were supposed to allow a communication between immune responses to different antigens and thus are fundamental for functioning of the idiotypic network (27). Needless to say, the sequential steps of the idiotypic cascade have also been detected in humans, e.g., during treatment of cancer patients with therapeutic antibody (53).

The reactivities in the idiotypic cascade allow two important conclusions to be drawn. First, although antigen-induced Ab1 can give rise to an anti-idiotypic response (Ab2) an immunization with Ab2 *never* induces the set of Ab1. This rule also applies for the next steps in the cascade. Hence, the idiotypic cascade only proceeds in a forward direction and not backwards. This is important since it has been concluded by Jerne (7) and others that “*recognizing*” and “*being recognized*” cannot be distinguished and that it is, therefore, meaningless to distinguish between idiotopes and combining sites. If this were the case, it could be argued that immunization with Ab2 should activate the whole set of antigen-induced Ab1. This, however, is not the case. Instead, Ab2 induces Ab3 that only in rare cases may contain antigen-binding Ab1′, which in addition are genetically different from Ab1. Second, immune responses to antibodies (Ab1, Ab2, and further) can only be induced after coupling to a carrier as, for instance, keyhole limpet hemocyanin that provides sufficient immunogenicity and with strong adjuvants and/or the help by different other means (54). Such efforts are absolutely inevitable for idiotypic/anti-idiotypic vaccinations of patients suffering from tumors or microbial infections (55, 56).

The extensive work on idiotypic regulation of adaptive immune responses allowed the statement “*that the immune system of a single animal after producing specific antibodies to an antigen, continues to produce antibodies to the idiotopes of the antibodies that it has itself made*” (7). This conclusion is based on the following principle idiotypic specificities and cellular interactions within a particular immune system [reviewed in Ref. (7, 57, 58)].

- The immune system contains anti-idiotypic B and T cell specificities for all antigen-specific BCR and TCR that have been investigated in this respect (29, 59).
- Idiotypic recognition occurs among B cells/antibodies (7, 27).
- TCR can be recognized by anti-idiotypic BCR/antibodies in an MHC-non-restricted fashion (60–63).
- BCR are recognized by anti-idiotypic TCR in an MHC-restricted way (64–69).
- MHC-restricted idiotypic recognition occurs among T cells (70, 71).

Despite these admittedly undeniable experimental evidences (72, 73) that were compiled in multiple experimental systems it was possible to state that “*it is safe to say that we never learned anything from it*” and that “*the idiotype network theory of regulation lacked logic and rationale*” (28) or to conclude that “*everything you can imagine to happen in the idiotypic network, you can make happen; but the physiological impact of these reactions remained elusive*” [K. Rajewsky in (29), p. 165]. Thus, a plethora of experimental results did not lead to a conceptual explanation as to why these interactions are induced. Were decisive experiments still missing? A solution to this key problem would have needed a clear comprehension about the principle nature of idiotopes as antigenic determinants that specifically characterize a particular idiotype.

## Characterization of Idiotopes

A particular BCR/TCR idiotype is composed of a collection of different idiotopes. Initially, idiotypes were characterized with absorbed xenogeneic and then allogeneic antisera before it became possible to properly identify individual idiotopes with monoclonal anti-idiotypic antibodies. However, anti-idiotypes of xenogeneic and allogeneic origin are generally broadly cross-reactive and thus are actually not idiotype-specific (74). Since a hypothetical physiological idiotypic network is exclusively functioning within a particular immune system (and at best between mother and fetus), autologous anti-idiotypes are actually mandatory for its investigation. Autologous anti-idiotypes have the highest discriminatory power and are truly specific for a given idiotope and therefore for the whole idiotype. For practical reasons syngeneic anti-idiotypes represent a reasonable compromise [reviewed in Ref. (74)]. During the entire research on idiotypic regulation, it could not be clarified whether idiotopes are non-inheritable individually specific characters or whether they are useful genetic markers. Thus, a generally applicable view of the nature of idiotopes in the BCR/TCR variable regions has not been presented. This is remarkable since the assembly of B and T cell antigen receptors from a set of gene segments in the primary lymphoid organs is well known. For a clear characterization of idiotopes, it seems advisable to briefly repeat the construction of BCR and TCR.

In contrast to the limited number of receptors and corresponding ligands, which, after clonally selected initiation of the response, mediate the further clonal development (activation markers, cytokines, interleukins, etc.) BCR/TCR are destined for and indeed able to recognize a seemingly unlimited number not only of environmental and autologous antigens but also of synthetic non-natural antigens. It is obvious that this task cannot be performed with a limited set of genomically encoded receptors. Thus, how can the immune system cope with this challenge? The generally accepted explanation is seen in the recombination of gene segments that together code for the variable domains of antigen receptors of BCR as well as both types of TCR exhibiting TCR_αβ_ or TCR_γδ_ receptors. All three are assembled from multiple V gene segments, diversity enhancing D gene segments, and J gene segments that join the variable portion with constant gene segments (75, 76). While variable regions of antibody light chains (V_L_), TCR_α_, and TCG_γ_ chains are assembled from V and J gene segments, those of antibody heavy chains (V_H_), TCR_β_, and TCR_δ_ chains are composed of V, D, and J gene segments. This V(D)J recombination allows already for huge hypothetical combinatorial BCR and TCR repertoires both of which, in addition, are greatly enhanced by inaccuracies that are introduced during the recombination process. However, a functional regulatory relevance (see below) has not been ascribed to these imprecisions which are somatically generated in three different ways.

(1) During V(D)J recombination, DNA hairpins are generated which may be opened asymmetrically. Thereby, a few nucleotides of one strand plus their complementary nucleotides from the reverse strand form a single-stranded tail. Filling-in of the second strand generates a palindromic sequence. In this way, a few nucleotides (P nucleotides) of the reverse strand are transferred to the coding sequence where they alter the information of the respective segment (75, 77–80).
(2) A quantitatively much more important modification at both recombination sites (V-D and D-J) of the BCR heavy chain (but only insignificantly at the light chain) as well as all four polypeptide chains of both types of TCR is created by the enzyme terminal deoxynucleotidyl transferase (TdT) that inserts non-templated nucleotides (N-nucleotides) at variable ratios (75, 81, 82). It has been estimated that TdT function is, for instance, at least responsible for 90% of the TCR_αβ_ repertoire (83). In contrast to the BCR V_L_ region, V-J-encoded TCR_α_ and TCR_γ_ chains also contain high numbers of N-nucleotides (76).
(3) Another equally important modification during BCR/TCR V(D)J recombination is introduced by exonuclease-mediated deletion of nucleotides from the 3′-end of V gene segments, both ends of the D gene segments and the 5′-end of the J gene segments (75, 76, 83).

**Figure 2:**
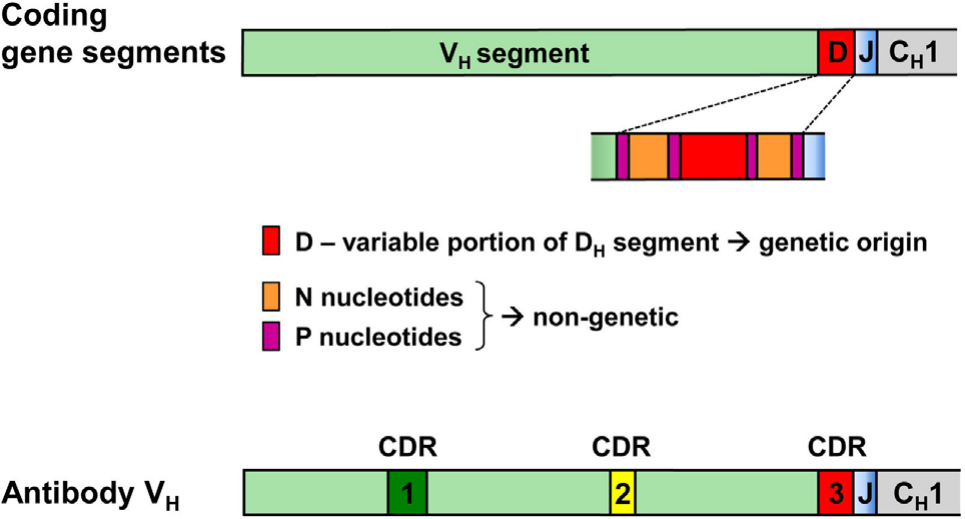
VDJ gene segments coding for the antibody heavy chain variable region and modifications creating the third complementarity-determining region CDR-H3 of antibodies. CDR-H1 and CDR-H2 are fully encoded in the genomic V_H_ gene segment. By contrast, CDR-H3 coding sequences are somatically created during recombination of V, D, and J gene segments. During this process, all three segments can be modified by deletion of nucleotides through exonuclease activity, addition of P nucleotides and insertion of N-nucleotides at the two junctions by terminal deoxynucleotidyl transferase. Hence, the D-region in the middle of CDR-H3 does not conform to genomic sequences and may be flanked by non-genetic N/P sequences that do not have an inheritable genomic counterpart.

**Figure 3:**
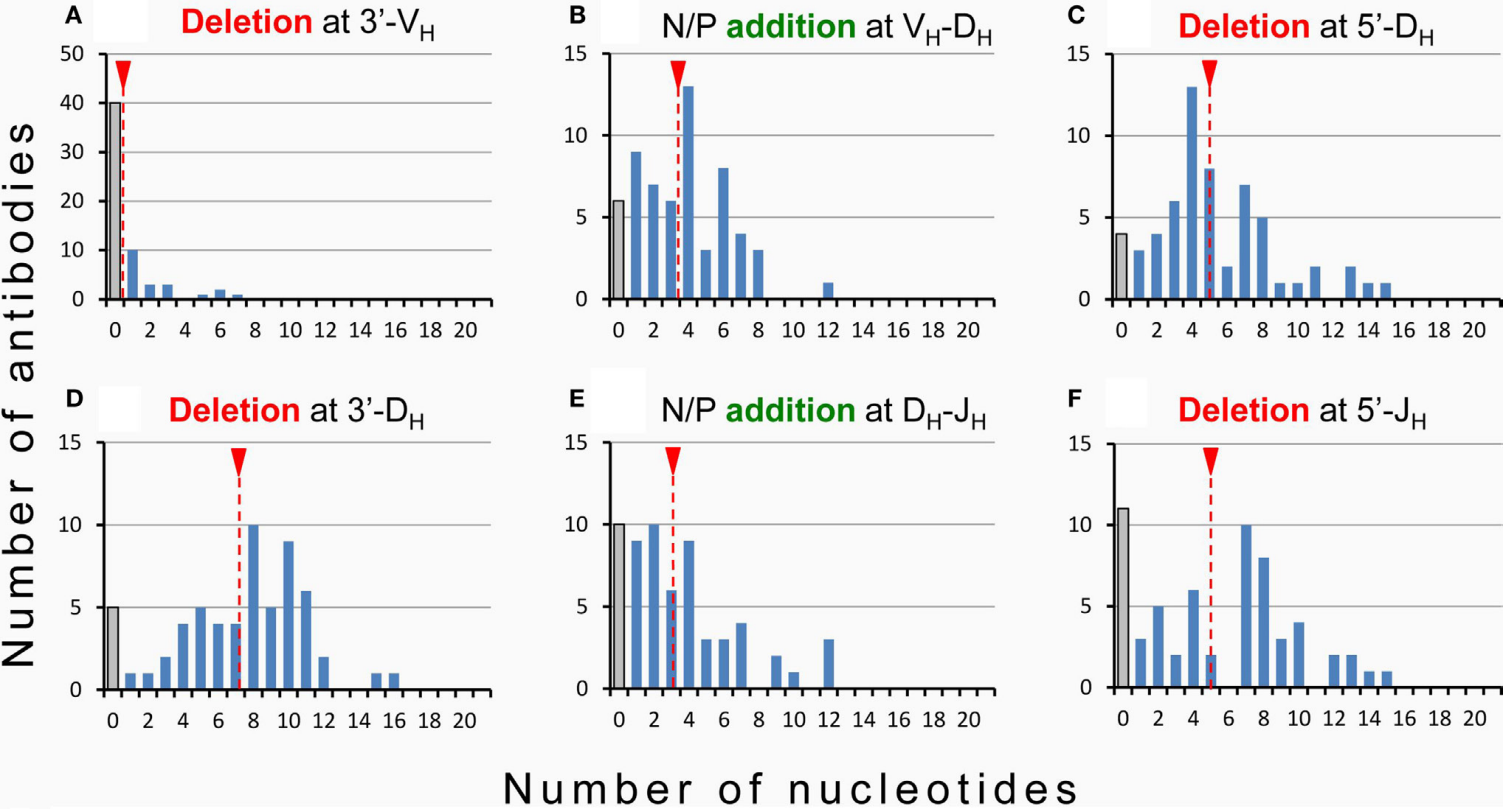
Modification of VDJ gene segments in anti-hapten antibodies. Homozygous mutant mice containing a single frameshifted DFL16.1 D gene segment (ΔD-DμFS mice derived from BALB/c mice) were immunized with the hapten 2-phenyl-oxazolone (phOx) coupled to chicken serum albumin (87). Monoclonal antibodies (*n* = 83) were prepared during various stages of primary and memory responses and V region sequences were determined (87). Fifty-eight (~70%) used modified D_H_ gene sequences and two mAb (~2.5%) reverse D_H_ sequences. Nucleotide deletion and addition of N/P nucleotides is shown for these two groups of antibodies: **(A)** nucleotide deletion at 3′-V_H_, **(B)** addition of N/P nucleotides at the V_H_-D_H_ junction, **(C)** nucleotide deletion at 5′-D_H_, **(D)** nucleotide deletion at 3′-D_H_, **(E)** addition of N/P nucleotides at the D_H_-J_H_ junction, and **(F)** deletion of nucleotides at 5′-J_H_. The medium values of deletion and addition are indicated by broken red lines with a red triangle on top. Antibodies using the genomic configuration at a particular site are represented by gray bars. In 23 additional mAb (~28%), the D_H_ coding sequence was truncated by exonuclease activity to such an extent that it could not be detected.

Hence, while the first two complementarity-determining regions (CDR1 and CDR2) of all BCR and TCR chains are fully encoded in the genome, the coding sequences of all CDR3s are somatically created. This is exemplarily outlined in Figure 2 for the VDJ recombination coding for the V_H_ domain of immunoglobulins. The genomic gene segments are modified by nucleotide deletions and additions of P and N-nucleotides to such an extent that the final products of CDR-H3 are specifically created for each B cell clone. Accordingly, CDR-H3 sequences are generally used as specific marker for the identification of particular B cell clones (26) and TCR CDR3 for T cell clones (84). Remarkably, the specificity of T cells is basically determined by TCR CDR3 with their almost unlimited variability (85). A quantitative impression of antibody CDR-H3 imprecisions is depicted in Figure 3 showing nucleotide deletions and additions of monoclonal antibodies that were obtained from BALB/c mutant mice containing a single altered D gene segment after immunization with chicken serum albumin coupled with the hapten 2-phenyl-oxazolone (phOx), one of the “classical” model immune responses (86) [data taken from Ref. (87)]. Although a certain proportion of these monoclonal antibodies exhibited neither deletions nor additions of nucleotides at both recombination sites V_H_-D and D-J_H_, none of the antibodies used a full genomic combination of VDJ gene segments. At both sides of the D segment up to 16 of 26 nucleotides could be deleted (Figures 3C,D) and 15 nucleotides at 5′-end of J_H_ (Figure 3F). What is even more remarkable, in 23 mAb (28%) the D_H_ sequence was truncated to such an extent that it could not be detected (data not shown). Similar results were obtained in another D-altered mouse strain (87). As we also did not find fully genomically encoded antibodies in a large group of phOx-specific antibodies from immunized as well as non-immunized BALB/c wild-type mice (23, 88), does this imply that there is no genomic VDJ combination with specificity for the hapten phOx?

**Figure 4:**
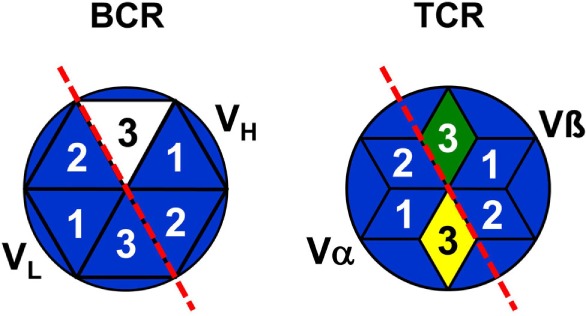
Representation of B and T cells by top views of their antigen receptors. B cells are schematically represented by stylized top views of their BCR paratopes as hexagons [based on Ref. (96)] and T cells by hexagonal stars [based on Ref. (97)]. Numbers indicate the complementarity-determining regions 1–3 of V_H_, V_L_, TCR_α_, and TCR_β_. BCR/TCR background colors symbolize the specificity of a particular paratope just as the genomic coding of CDR1 and CDR2. The full nonself-quality of CDR3s is indicated by different colors, but the marginal nonself-character of CDR-L3 is not shown.

In addition, it has to be asked as to whether antibodies exist at all, which are encoded by complete genomic V, D, and J gene segments without recombination-introduced inaccuracies as the assumption of a combinatorial repertoire suggests? Antibodies as products of antigen-stimulated adaptive immune responses are produced by “conventional” or so-called B-2 cells (89). To the best of my knowledge, in repertoire analyses of B-2 cell antibodies of adult mice full genomic sequences have not been found as exemplarily shown in Ref. (90–92) as well as in a large collection of sequences of anti-phOx antibodies (23, 87, 88, 93). In contrast to B-2 cell antibodies, nAbs are produced and secreted by B-1 cells without stimulation by external antigens. B-1 cells are basically subdivided into CD5-expressing B-1a and CD5-negative B-1b cells (89) and their products of nAbs constitute a heterogeneous group of antibodies (94). Due to the lack of TdT in early ontogeny, sequences of B-1 cell-derived nAb from fetal and neonatal mice do not or very rarely contain N-nucleotides (90). However, this proportion increases with age so that two-thirds of adult B-1a transcripts contain N-nucleotides and show a considerable CDR-H3 diversity (92). It has been argued that the fraction of N-nucleotide-negative nAbs are *therefore* completely germline encoded (95). This conclusion, however, is challenged by sequences of neonatal nAbs (as well as TCR_γ/δ_) that, although almost devoid of N-nucleotides, are *not* encoded by fully genomic VDJ combinations (90–92) since variable numbers of nucleotides are deleted from 3′-V_H_, both sides of D_H_ or 5′-J_H_ sequences. Thus, as none of these nAbs and TCR_γ/δ_ represents the full genomic information (90) it can be concluded that CDR3s of practically all BCR, TCR_α/β_, and TCR_γ/δ_ are somatically created and *not* fully derived from pure genomic sequences. This is related to the problem of self–nonself-discrimination which is certainly not determined in the genome but depends on genomically encoded translated products. Hence, with all likelihood, CDR3s of all antigen receptors belong to the nonself (Figure 4).

This is of fundamental importance for two reasons [reviewed in Ref. (58)]:

(1) Although generally ignored, there is multiple evidence that antigen-activated B and T cells not only present antigen-peptides on MHC molecules but also peptides of their own intrinsic antigen receptors which can be recognized by other anti-idiotypic T cell clones contained in the normal repertoire (66, 68, 69, 71, 98–100).
(2) Various experimental investigations as well as clinical observations have clearly shown that CDR3-associated antigen receptor-intrinsic nonself-portions of non-mutated BCR and TCR represent the autologous clone-specific or idiotypic characters of antigen receptors. In BCR, in addition, new and thereby nonself-idiotopes are created and accumulate during TD immune responses by SHM (84, 101–109).

## Immunogenicity of Autologous Idiotopes

The question is now whether adaptive immune response-initiating BCR and TCR, because of their nonself-portions, are competent to induce anti-idiotypic responses by themselves as suggested in the idiotypic network theory (7, 57, 110). Herein, Jerne supposed that, before any engagement in immune responses to foreign antigens, the size and state of activity of B (and T) cell clones is regulated by reactivity of their inherent idiotopes with other anti-idiotypic B (and T) cells contained in the system (27). Thus, the immune system was viewed as primarily self-centered and would strive in its entirety to reach a dynamic equilibrium of mutually interacting B and T cell clones. Jerne concluded that the antigen-free (AgF) immune system displays an “eigen”-behavior that results from idiotope–paratope interaction within the system and that “*the invasion into our body of foreign particles, proteins, viruses, or bacteria, (which) incidentally disturb the dynamic harmony of the system*” (7). However, this view did not remain uncontested. As the antigen-independent differentiation in the primary organs results in a unique cell, and not a clone of cells that can be activated by antigen as the CSL implies, Paul and Bona (111) argued that concentrations of idiotope and its corresponding anti-idiotypic paratope and their chance to encounter each other are too low to induce a functional connection. Hence, they argued that an idiotypic network that is endowed with an *eigen*-behavior does not exist before stimulation by antigen. The same conclusion can also be drawn from the following investigations. Since idiotopes represent TD antigens it has to be expected that such responses would lead to isotype-switched IgG anti-idiotypes. An answer to the question as to whether idiotopes are *per se* immunogenic can be deduced from studies of experimental animals whose microbial load is stepwise diminished. Compared to conventional mice the serum concentrations of all immunoglobulin classes except IgM is significantly lower in specific pathogen-free mice (112) and further decreased in germ-free (GF) mice (113) and virtually absent in AgF mice [reviewed in Ref. (114)]. Strikingly, the reduction of humoral immunoglobulins correlates with a severe impairment of many parameters of the innate immune system as well as the whole T cell compartment (115, 116). Consequently, numbers of CD4^+^ T cell subpopulations, CD8^+^ memory T cells, various innate lymphoid cells, blood cell gene expression patterns, resistance to infections, as well as humoral immunoglobulins have been found to increase to the state of conventional mice when such “clean” mice are re-infected with known collections or commensal microbes (117, 118). Thus, since sera of AgF mice are virtually devoid of IgG antibodies it can first be concluded that humoral IgG fully depends on stimulation by environmental antigens and second that antigen receptor-intrinsic nonself-portions are *per se* apparently non-immunogenic.

## Evidence for Physiological Impact of Autologous Idiotopes

The idiotypic network theory does not include a clear distinction between the antibody’s antigen combining site, the paratope, and its contained idiotope(s), and certain experiments were taken as evidence for a potential complete overlap between the combining site and binding of anti-idiotypes (7, 27). This led to the assumption that the shape of paratopes of particular anti-idiotypes represent internal images of the corresponding antigenic epitopes and that both BCR and TCR repertoires, therefore, contain internal images of all antigenic determinants of environmental antigens (see above) (7, 27). However, it is crucial to clearly discriminate between the dimension of BCR and TCR antigen combining sites that are formed by the six CDRs (96, 97) and the intrinsic nonself-idiotopes as BCR/TCR parts that are recognized by other anti-nonself B and T cells contained in both autologous repertoires, whereas reactivities of xenogeneic and allogeneic anti-idiotypes are misleading. Hence, the recognition that antigen receptor-associated nonself-portions constitute the idiotypic characters of BCR and TCR reject the view of internal image antibodies. Instead, “internal image” activity depends on idiotypic cross-reactivity so that anti-idiotypes may recognize idiotypes of completely different antigen-specificity (see above). These considerations document a clear and, at least in principle, facile explanation for idiotypic cascade-like chain reactions that are induced during antigen-induced immune responses, which contribute immunogenicity and adjuvanticity [reviewed in Ref. (58)]. This allows for a unification of clonal selection and idiotypic network theories, which hitherto are believed to be incompatible (28, 29). Hence, the recognition of antigen receptor-intrinsic nonself-portions as idiotypic characters prove that idiotypic regulation represents a physiological necessity. This view contributes to explain a variety of hitherto incomprehensible experimental findings:

(1) Contrary to the CSL, the immunization with many viruses, bacteria, and protein antigens not only leads to a specific response to these antigens but also simultaneously to a much higher increase of total immunoglobulins (18, 26, 119). This immunization-associated polyclonal B cell activation is not only brought about by a variety of non-specifically acting factors including mitogens and cytokines that are secreted by activated T cells [discussed in Ref. (119)]. There are also indications that this polyclonal immunoglobulin production (including IgG) is not totally antigen-non-specific since simultaneous immunization with two antigens demonstrated that each antigen induced its own specific and non-specific response, i.e., both effects were additive (120). This antigen-related effect was supposed to be activated by a second BCR-specific and thus idiotype-specific helper mechanism (121).
(2) Although idiotypic nonself-portions of antigen receptors are *per se* non-immunogenic, as can be deduced from experiments in AgF animals (see above), autoantibody-masking and suppressing anti-idiotypic antibodies are frequently induced in healthy individuals containing a normal commensal microbial flora (122, 123).
(3) Although numerous FDA-approved therapeutic antibodies have successfully been used and seemingly never caused fatalities, but only mild infusion reactions that could easily be managed, it has also been reported that the clinical use of therapeutic antibodies of murine origin has been hampered because of occasionally severe side-effects that could be ascribed to their strong CDR-associated immunogenicity. Therefore, great efforts have been undertaken to reduce this immunogenicity by humanization and finally by use of human antibodies. However, in a certain percentage of patients, even fully human therapeutic antibodies may still produce pathological side effects through the induction of human anti-human antibodies (124, 125) that are directed at CDRs (126–128).
(4) Besides its secondary effector functions, humoral immunity can actively stimulate regulatory inductor functions [reviewed in Ref. (58)]. This is of particular importance for the transfer of the immunological experience of the mother to the newborn that aids the initial development of the nascent immune system and induces long-term effects (129). In various experimental systems, it has been demonstrated that immune as well as maternally derived monoclonal antibodies induce an immunological imprinting that alters the immune responses to the respective antigens for life-time (130, 131). Therefore, F1 and even F2 offspring of immunized dams cannot be regarded as “normal” mice when maternal antibodies are not any longer detectable. Importantly, these maternal effects can be induced with maternal antigen-reactive antibodies (idiotypes) just as their corresponding antigen-non-reactive anti-idiotypes as demonstrated, for instance, for protection against microbial infections with respiratory syncytial virus (132) or group B streptococci (39).
  Moreover, maternal antibodies selectively suppress IgE isotype responsiveness to antigens experienced by the mother (133). In an experimental model of food allergy to ovalbumin (OVA), the transgenerational IgE suppression by maternal OVA-containing immune-complexes (OVA-IC) conferred long-lasting protection against food anaphylaxis that was assumed to be mediated by OVA-specific Foxp3^+^ Tregs (134). However, this conclusion is questionable for three reasons. (i) The supposed OVA-specificity exclusively rests on the observation that Foxp3^+^ Tregs from mesenteric lymph nodes of offspring of OVA-sensitized dams proliferated stronger upon stimulation with OVA *in vitro* as compared to Tregs from non-sensitized dams or stimulation with peanut extract as irrelevant antigen. This conclusion would be valid if antigen stimulation would lead to exclusive activation of antigen-specific clones, as proposed in the CSL. This, however, is not the case because of the polyclonal nature of the response that includes the idiotypic chain reaction (see above). (ii) In addition, antigen-non-reactive suppressor T cells have been demonstrated in a similar experimental setting. Offspring of OVA-immunized female rats showed a transient suppression of IgG and IgM responses but a persistent OVA-specific IgE suppression, which was mediated by CD8^+^CD4^−^ T suppressor cells although these cells did not react with OVA (135). (iii) In the work of Ohsaki and co-workers (134), only maternally derived OVA-IC are taken into account thus emphasizing a central role of the antigen in mediating the transgenerational IgE suppression while a major functional impact of OVA-specific IgG antibody is not considered. However, this is necessary since it has been shown that a transgenerational suppression of IgE responsiveness to bee-venom-phospholipase A_2_ (bvPLA_2_) allergen can also be achieved in a completely antigen-free experimental system with monoclonal antibodies (136). Maternally derived mAb not only suppress the parenteral IgE response to bvPLA_2_ but also IgE responses that are induced by airway-immunization with nebulized ovomucoid-containing OVA (137). This suppression lasts until an age of 4 months but is not any longer detectable at 6 months. However, when IgE-inducing immunizations were started before an age of 4 months and continued in monthly intervals IgE suppression persisted for more than a year (137). This experimental finding is in line with considerations that early encounter of allergens might have a protective effect on later development of allergies (134, 138) which even may override a genetic predisposition (137, 139). In addition, in full agreement with the idiotypic chain reaction, even the postnatal transfer *via* colostrum and milk of a monoclonal anti-idiotypic mAb reactive with an IgG-anti-PLA_2_ mAb induced long-lasting IgE suppression while leaving the IgG response unaltered (140).
(5) An inductor function of antibodies has also been demonstrated in various experimental systems showing a mutually dependent development of both BCR and TCR repertoires. For instance, B cell-deficient mice exhibit a drastically reduced T cell repertoire the restoration of which could be achieved with immunoglobulin preparations or B cells in an Fc-independent manner (141, 142). Strikingly, this regeneration did not depend on the amount but on the diversity of the applied immunoglobulin and B cells. These observations acknowledge earlier studies, which had shown that the generation of B and T cell repertoires is mutually dependent (143–145).
(6) Interclonal idiotypic regulations among B and T cells are clearly visible during vaccination of autoimmune or malignant diseases with auto- or tumor-antigen-reactive T cells [T cell vaccination (TCV)] (146, 147). In accordance with the idiotypic chain reaction, however, a successful TCV depends on the use of activated T cells, which not only induce anti-idiotypic responses to TCR CDR3 idiotopes but also anti-ergotypic responses, which are directed at activation markers of the inducing effector T cells irrespective of their TCR specificity (84). In addition, TCV-induced immunity needs the participation of B cells (148, 149). Effective TCV has been demonstrated in a variety of experimental animal models and clinical trials have provided promising results for the treatment of multiple sclerosis and have shown some clinical improvement in mild form of systemic lupus erythematosus (150).

Hence, immune responses in general induce subsequent idiotypic interclonal connections. These findings require reconsideration of the content of immunological memory, which is apparently not restricted to antigen-specific B and T cell clones but comprises a net of many idiotypically interconnected clones. Is it possible to correlate these idiotypic connections with current research into the regulation of adaptive immune responses?

## Post-Initiative Involvement of Idiotypic Regulation of Immune Responses

Selection of clones with higher affinity (immune maturation) has long been assumed to be the main driving force for clonal progression during the immune response. In the T_FH_ cell-depend GC response, somatically mutated B cells with the highest affinity are expected to preferentially concentrate and present antigen and thereby attract more help from T_FH_ cells for further differentiation and clonal selection during class-switching. However, this simple scheme had to be modified since the amount as well as the activity of T_FH_ cells also depends on a specialized type of follicular Foxp3^+^ regulatory T cells (T_FR_) [reviewed in Ref. (24, 151)]. Although T_FR_ cells phenotypically resemble T_FH_ and extrafollicular Tregs they constitute an own subpopulation that originates from thymic Foxp3^+^ precursors and limit numbers of T_FH_ cells and GC antigen-specific as well as antigen-non-specific GC B cells (152). T_FR_ cells inhibit GC reactions, affinity maturation just as plasma cell (PC) differentiation (153). Moreover, dampening of the GC reaction by Foxp3^+^ T_FR_ cells reduces the amounts of secreted antigen-specific IgM, IgG1, IgG2b, and IgA (154). It is of interest that after immunization with the TD antigen sheep red blood cells (SRBC) splenic T_FH_ and T_FR_ cells (ratio ~100:16) develop with different kinetics. Highest numbers of T_FH_ are observed between days 7 and 11 while T_FR_ cell numbers subsequently peak during days 11–17 (24). Since the SRBC-specific share of antibody-secreting cells in this response is only ~10% compared to ~90% of PCs secreting immunoglobulins of unknown specificities (121) it seems to be conclusive that the involved T_FH_ as well as T_FR_ cells also represent heterogeneous populations with highly diverse repertoires. T_FR_ cells may exert their regulatory function by direct cell–cell contacts with B and T cells (or indirectly *via* secreted cytokines) (24). A mechanism for this action, however, is not known and would probably rest on the determination of T_FR_ specificities. As the idiotypic nonself-portions of all antigen receptors have hitherto not been taken into account it is tempting to speculate that components of the idiotypic cascade are at least part of the T_FR_ repertoire.

Thus, are there direct indications that the GC response and in particular its regulation by T_FR_ cells might depend on idiotypic interactions? Already during the heydays of research on idiotypic regulation, it was speculated that “*a very dominant negative selection, perhaps against the idiotype of primary antibodies*” might be involved in clonal progression during the immune response (155). Such an idiotype- resp. CDR-H3-directed regulation gained support by several later investigations demonstrating that other factors than affinity-selection must be involved [reviewed in Ref. (58)]. For instance, already 1 week after immunization the first GC memory cells, even when unmutated, express shorter idiotypic CDR3 in the V_H_ region (CDR-H3) than antibody-secreting PCs and, by the end of the second week, most clones exhibiting affinity-enhancing mutations were also characterized by shorter CDR-H3 (156). A correlation between frequency of mutations and shorter lengths of CDR-H3 has also been observed in human antibodies (157, 158). In the murine model immune response to the hapten phOx the dominance of a particular idiotype (Id_Ox1_) is T cell-dependent and first established during class-switch-recombination; concomitantly, CDR-H3 diversity is drastically reduced and accompanied with shortening and approximation of CDR-H3 lengths and with elimination of IgM-secreting clones despite their equal or even considerable higher affinities for the hapten than Id_Ox1_ antibodies (88). Thus, contrary to previous understanding, the Id_Ox1_ dominance does not seem to depend on its superior affinity but appears to be idiotypically selected. An idiotypic selection of slightly shorter and more uniform CDR-H3 lengths during class switching is also indicated in the response of C57BL/6 mice to the hapten NP (88, 159). Hence, these findings suggest the involvement of BCR-specific, namely idiotope-/CDR-H3-specific T cells during CSR and subsequent clonal selection in GCs.

Direct evidence for an idiotypic regulation has been provided in a murine adoptive transfer model showing that BCR peptide-specific (thus idiotype-specific but antigen non-reactive) CD4^+^ T cells interrupt the GC reaction, inhibit the secondary response, and redirect the differentiation of B cells into extrafollicular plasmablasts (160). Furthermore, a TCR-idiotype-specific regulation of immune responses has also been demonstrated for CD8^+^ T cells that are restricted by the MHC class Ib molecule Qa-1 (HLA-E in humans), which is absent from naïve resting CD4^+^ T cells but transiently expressed after antigen activation (106). Blockade of this regulatory pathway in Qa-1-deficient mice leads to enhanced responses of CD4^+^ T cells to foreign as well as autoantigens (161).

## Concluding Remarks

**Figure 5:**
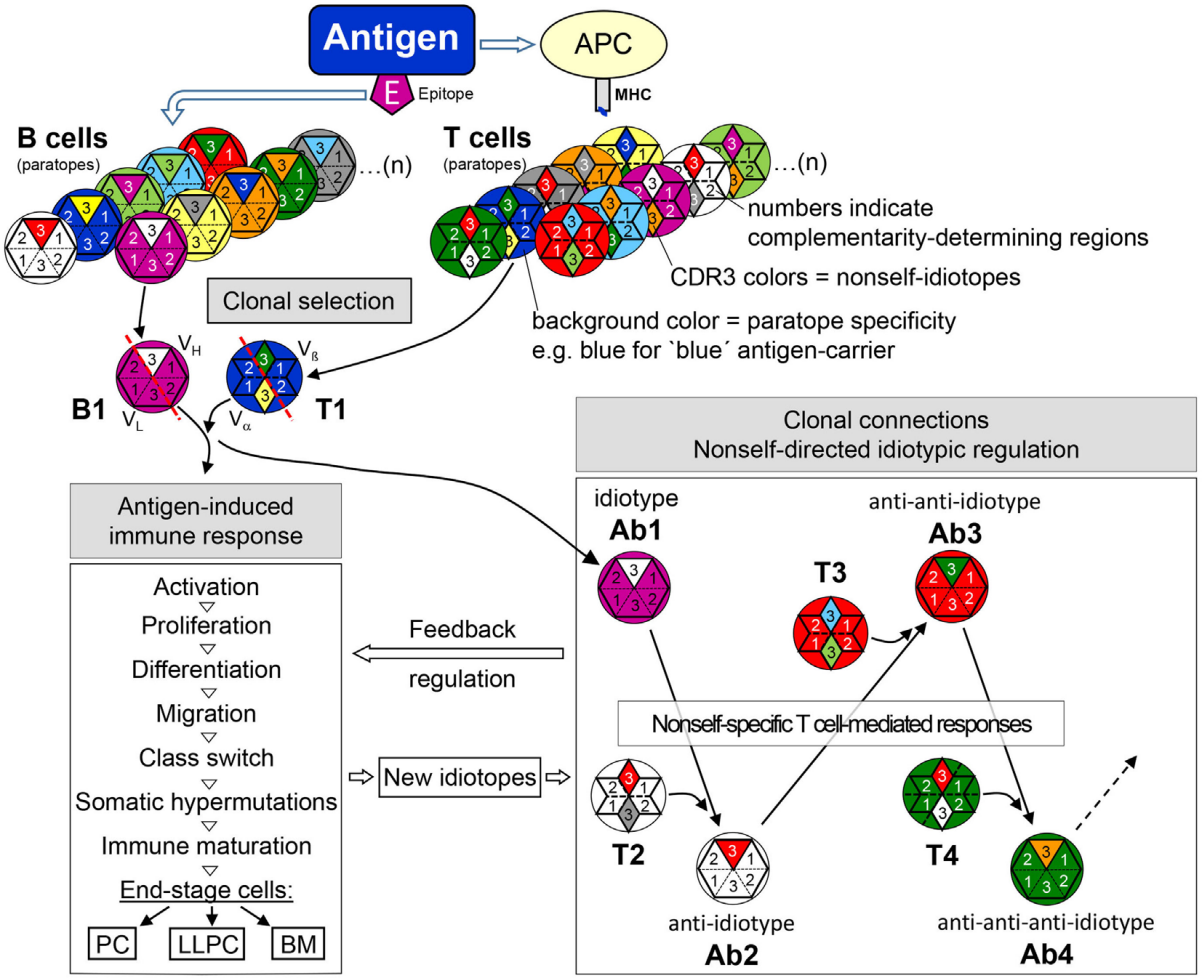
The adaptive immune response—from clonal selection to nonself-directed idiotypic regulatory clonal connections. Antigen selects epitope-specific B cells and carrier-specific T cells from the respective repertoires. BCR/TCR background colors symbolize the specificity of a particular paratope (see legend to Figure 4), in this example, purple for the B cell epitope-specificity and blue for the carrier-specificity of T cells. After antigen-induced activation, CDR-H3 nonself-idiotopes (white) of antigen-specific idiotype (B1/Ab1—purple paratope) activates nonself-specific anti-idiotypic B cells (Ab2—white paratope) with the help of likewise nonself-specific anti-idiotypic T cells (T1—white paratope). In further reaction, nonself CDR-H3 portions of Ab2 (red) may be recognized and activate anti-anti-idiotypic B cells (Ab3—red paratope) with the help of Ab2-idiotype-specific T cells. This idiotypic cascade may proceed to anti-anti-anti-idiotypes (Ab4) and even further as shown for Ab6 (162). Thus, clonal selection and activation by antigen induces clonal connections of an idiotypic cascade that exerts a regulatory feedback on the ongoing antigen-induced response. During this response, in addition, somatic mutations create new nonself-idiotopes which may also induce an idiotypic feedback regulation. Abbreviations: APC, antigen-presenting cell; MHC, major histocompatibility complex; PC, plasma cell; LLPC, long-lived plasma cells; BM, B memory cell.

As receptors generally exert exclusive specificity for their genuine ligands it is taken for granted that BCR/TCR, because of their name “antigen receptors,” exclusively bind antigens resp. MHC-presented antigenic peptides. It was almost regarded as a sacrilege that BCR/TCR should perform meaningful reactions with other autologous anti-idiotypic antigen receptors. Thus, over time, the sole reactivity with antigens became a firmly established matter of course. The intrinsic reactivity with anti-idiotypic BCR/TCR did not make sense since it seemingly discredited the clear view that the immune system’s task is the fight against microbial infections. This view, however, is not any longer tenable since idiotypic characters that are distinguishable in the autologous immune system are recognized as BCR/TCR nonself-portions in association with all CDR3s (but only slightly with that of the immunoglobulin L-chain) and somatic mutations in the variable regions of BCR that are generated during the adaptive immune response. As the self–nonself-discrimination is the fundamental question of all immunological research, immunology as a discipline has even been viewed as the science of self–nonself-discrimination. Hence, it is incomprehensible why CDR3 sequences although not encoded in the genome but somatically created and used as clonotypic markers, have not been recognized as idiotopes that belong to the nonself. Although foreign, these nonself-portions are non-immunogenic by themselves as can be concluded from experiments with AgF animals. However, antigen activation is the initial spark for *clonal selection* and cellular proliferation that is accompanied with an increase of antigen receptors plus their nonself-portions and expression of activation markers. Thereby, idiotypic (as well as ergotypic) characters become immunogenic and initiate further anti-idiotypic (and ergotypic) *clonal connections* that form a waning idiotypic chain reaction as schematically depicted in Figure 5. Hence, as proposed in the idiotypic network theory (7), the activation of adaptive immune responses is followed by regulatory idiotypic chain reactions. However, just as the idiotypic network theory by its own could not give a complete and satisfactory understanding of the adaptive immune response this cannot be expected from sole observance of the function and sequential expression of non-specific markers (lineage and activation markers, transcription factors, cytokines and their receptors). This can only be achieved by investigating the interdependency of clonotypic activation and idiotypic regulation both of which represent initializing regulatory principles that subsequently lead to expression of non-specific cellular markers and cytokine/chemokine secretion driving the differentiation of lymphocytes during antigen/autoantigen-induced immune responses. In conclusion, the well-documented fact of the autologous recognition of antigen receptor-intrinsic nonself parts as targets for idiotypic regulation of adaptive immune responses needs to be incorporated in future investigations.

## Author Contributions

The author confirms being the sole contributor of this work and approved it for publication.

## Conflict of Interest Statement

The author declares that he has neither commercial nor any financial relationships that could be construed as a potential conflict of interest.
